# Association between recipient survival and blood donor age after blood transfusion in a surgery intensive care unit: a multicenter randomized controlled trial study protocol

**DOI:** 10.1186/s13063-020-04452-6

**Published:** 2020-07-08

**Authors:** Xianfei Zeng, Yan Liao, Xiaoshuang Wu, Jinmei Xu, Chenxing Da, Zhijun Tan, Fan Feng, Wen Yin, Dongjian Wang, Xingbin Hu

**Affiliations:** 1 School of Medicine, Northwest University, Xi’an, 710069 China; 2 The National Engineering Research Center for Miniaturized Detection Systems, College of Life Science, Northwest University, Xi’an, 710069 China; 3 Department of Transfusion Medicine, Shaanxi Corps Hospital, Chinese People’s Armed Police Forces, Xi’an, 710054 China; 4 Department of Transfusion Medicine, Third Affiliated Hospital of Guangxi Medical University, Nanning, 530031 China; 5 Department of Transfusion Medicine, Xijing Hospital, The Fourth Military Medical University, Xi’an, 710032 China; 6 Department of Endocrinology, Shaanxi Corps Hospital, Chinese People’s Armed Police Forces, Xi’an, 710054 China; 7 Department of Statistics, Fourth Military Medical University, Xi’an, 710032 China; 8 Department of Digestive Surgery, Xijing Hospital, Xi’an, 710032 China; 9 Department of Transfusion Medicine, 908th Hospital of PLA, Yingtan, 335000 China

**Keywords:** Blood transfusion, Critically ill, Blood donors, Randomized controlled trial

## Abstract

**Background:**

Blood from younger individuals has been shown to improve physiological function in recipients in laboratory research, and many proteins from human peripheral blood show antisenescence capabilities. Thus, researchers have questioned whether blood from young donors is superior to blood from older donors. Blood transfusion is a key supportive therapy for trauma patients, and recent studies have reported the influence of blood donor age on recipient patient prognosis. Although some retrospective results found that blood from young donors improves survival, no influence of blood donor age was observed on outcomes in other study groups. The reasons for this discrepancy are complicated, but the fact that data were not obtained from randomized controlled trial (RCT) data should be considered. The current protocol and analysis method provide a feasible RCT design to evaluate the prognosis of severely ill surgery patients who were transfused with blood products from blood donors of different ages.

**Methods:**

The current study is a pragmatic multicenter RCT (open, parallel-group, non-masked, superiority trial). Recruited surgery intensive care unit patients will be randomized into three groups and transfused with blood products from male donors of different ages (< 25, 25–45, and > 45 years). Survival time will be measured within 28 days. The survival characteristics, possible interaction between variables, and potential factors associated with death will be analyzed by Kaplan–Meier analysis, two-way ANOVA, and Cox proportional hazards model, respectively.

**Trial registration:**

ChiCTR: ChiCTR190002. Registered on 22 March 2019. http://www.chictr.org.cn/showproj.aspx?proj=36867.

## Background

Mass trauma and major surgery cause extensive blood loss, coagulation disorders, infections, and inflammatory responses. These factors contribute to high mortality due to their complicated therapies. Identifying methods to decrease patient mortality in surgery and trauma units is an important issue for hospital facilities, including the transfusion department.

In fact, blood transfusion is a key supportive therapy for mass trauma or surgery patients. But recently, several overlooked donor factors have been reported to affect the prognosis of blood recipients. These factors include donor sex and age, and they impose great challenges to classical blood transfusion strategies [1–5].

Heterochronic parabiont animal studies showed that blood from young donor mice improved several age-related functions of older mice, such as cognitive capability, hepatocyte proliferation, cardiac hypertrophy, and bone remodeling [6–11]. In blood, the plasma protein growth differentiation factor 11 (GDF-11) [7, 12] and mesencephalic astrocyte-derived neurotrophic factor (MANF) have been shown to be beneficial for aged individuals. Notch and Wnt signaling are also involved with improvements in aging [13, 14]. These observations could form the basis for a novel theory on reversing aging in humans.

Since proteins in plasma have been suggested to have anti-senescent effects [11], several researchers attempted to study patients immediately after blood transfusion. Thus, several reports on the impact of blood donor age on patient prognosis have emerged in recent years. Researchers stated that an increased risk of death existed among recipients of blood from young and female donors when a complex, time-dependent survival model was used [5, 15]. However, the opposite finding has been observed in other groups; a cohort study using data extracted from the Scandinavian Donations and Transfusions database concluded that the age and sex of blood donors did not influence patient survival, which has been further supported by other studies [3, 16]. Furthermore, a meta-analysis mentioned that age, sex, and blood type played a role in recipient prognosis after blood transfusion [17]. No consensus currently exists on whether blood from younger donors can improve the rehabilitation and survival of recipient patients. In addition to differences in the design, methods, and sample sizes, these data are often from retrospective studies. Therefore, more detailed clinical research, particularly from RCTs, is urgently needed. However, in contrast to other RCTs, performing an RCT on this issue is difficult due to the limited shelf-life of blood products and to multiple blood transfusions with mixed blood types.

Two studies are in progress to evaluate the effect of young donor blood in patients with Alzheimer’s disease (NCT01561053 and #NCT02256306), but the outcomes are not available yet. To assess the donor age influence on prognosis (DAIP) of severely ill patients in mainland China, we designed the current protocol and will recruit patients from a surgical intensive care unit (SICU). With rational inclusion and exclusion criteria, patients from different medical centers will be enrolled in the present study. After randomizing and blinding design and data collection have occurred, the 28-day survival will be taken as the primary outcome, to evaluate the influence of younger donor blood transfusion on patients.

## Methods/Design

**Fig. 1:**
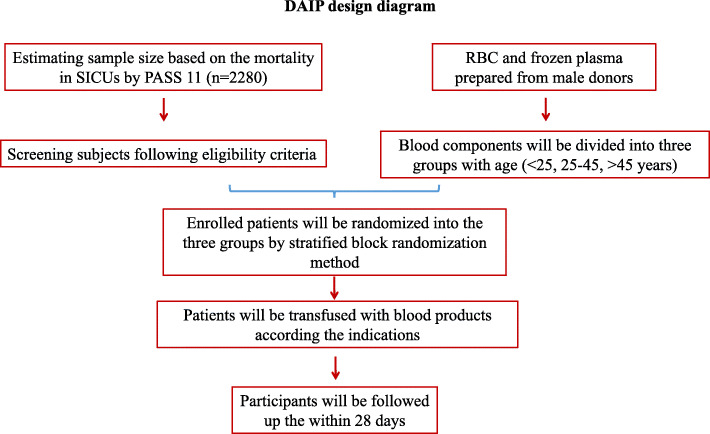
DAIP design frame. *PASS* software for sample size evaluation, *SICU* surgical intensive care unit. Blood components in the DAIP study will include red blood cells units and plasma

**Fig. 2:**
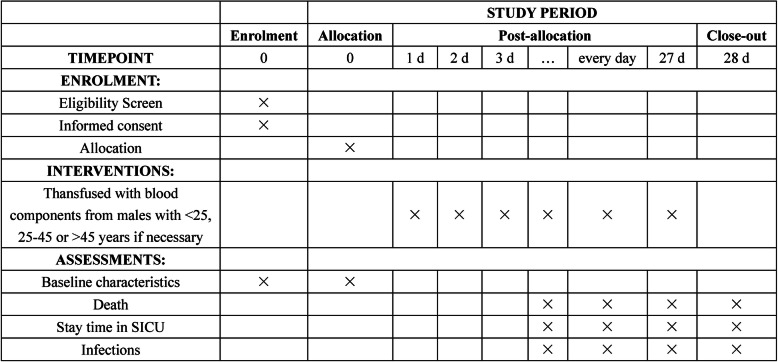
The schedule of enrollment, interventions and assessments demonstrated in the Standard Protocol Items: Recommendations for Interventional Trials (SPIRIT) Figure

All the design and analysis procedure will be executed as shown in Fig. 1. The SPIRIT checklist (see Additional file 1) guided our protocol development. The SPIRIT figure reflecting the participant timeline and study protocol are presented in Fig. 2.

### Study objectives

#### Primary objectives

The primary objective of the proposed study is to assess differences in 28-day survival between SICU patients who were recipients of blood transfusion from younger or older donors.

#### Secondary objectives

Patient stay time in the SICU and infection rates in recipients who were transfused with blood components from younger or older donors will be recorded. Results from an Acute Physiology and Chronic Health Evaluation (APACHE-II) and Sequential Organ Failure Assessment (SOFA) will be also stored during this DAIP research.

### Eligibility criteria and intervention

The proposed study is an RCT. The research results will provide data to test the hypothesis that blood transfusion from young donors benefits recipient patients. The study will be performed in three medical centers.

#### Eligibility criteria

Inclusion criteria. (1) Patients with trauma in the SICU with homologous blood transfusion and (2) patients undergoing major surgeries with a predicted volume of blood transfusion greater than 1000 mL (RBC + frozen plasma + cryoprecipitate) will be included.

Exclusion Criteria. (1) Patients aged < 18 or > 70 years, (2) patients with hematopathy, (3) patients requiring chronic blood transfusions, (4) patients with cancer who are undergoing conservative treatment, (5) patients who were not enrolled in the study before being admitted to the SICU and had undergone homologous blood transfusion, (6) patients with pre-existing pulmonary or bloodstream infections before being admitted to the SICU, (7) patients who were transferred to a general ward after fewer than 3 days stay in the SICU, (8) patients with autologous blood transfusion, (9) patients receiving hematopoietic stem cell transplantation, (10) patients receiving solid organ transplantation, and (11) patients who received a transfusion with single-donor platelets in which the donor age was inconsistent with the donor age of the previous blood components will be excluded.

#### Interventions

After randomization into three groups, the enrolled patients will be infused with blood from male donors of different ages (< 25, 25–45, and > 45 years). Blood components including red blood cells (< 14 days old), frozen plasma, and cryoprecipitate for transfusion will be acquired from Xi’an Blood Center of People’s Liberation Army (PLA), Yingtan Blood Center, and Blood Center of Guangxi Province. Blood will be processed and prepared by a local blood center according to their standard protocols. The indications for blood transfusion will follow the requirements of Industry Standards for the Healthcare Industry (WS/T 622–2018 and WS/T 623–2018).

Implementing the blood allocation principle designed in DAIP investigation will not require alteration to usual care pathways, and it will continue for both trial arms.

### Determination of sample size

Twenty-eight days mortality is the most significant indicator in the study design, as it directly mirrors the recovery of SICU patients. The sample size will be statistically estimated based on 28-day mortality data from three centers in 2018, of which 15% is estimated to reflect SICU patients undergoing blood transfusion. We assume that the transfusion of blood from young donors will improve the survival of SICU patients, and we estimate a significant statistical difference of 3% mortality change between groups. Therefore, the mortalities of patients in each of the three groups are predicted to be 12%, 15%, and 18%, respectively. We performed a chi-square test with proportion difference to estimate the sample size, assuming an alpha of 0.05 and power of 80%. Considering the loss to follow-up, which is set at 10%, the adjusted sample size is 2276 cases. Therefore, we plan to include 2280 cases in the study. This trial will be carried out in three centers, and subjects will be allocated to centers at a ratio of 2:1:1. The three centers of Xi’an, Nanning, and Yingtan will require 1140, 570, and 570 subjects to be enrolled, respectively.

### Recruitment and ethics claim

The patients will be recruited following the eligibility criteria. To enable us to reach the sample size, the awareness rate of the research innovation and significance by surgeons, patients, and their relatives will be raised by lectures and instant messages to emphasize the potential role of biological factors for rejuvenation or regeneration in younger blood. We estimate that the sample size will be reached after approximately 20 months, based on the number of SICU patients in the three centers. Written informed consent will be obtained from all patients enrolled. The informed consent of unconscious participants can be given and signed by their first-degree relatives. In each center, an authorized research nurse in the clinical unit and a technician in the Department of Blood Transfusion will be responsible for recruiting the patient. The nurse will talk to the patient to gain informed consent. Participants will be asked if they agree to the use of their data, and they can choose to withdraw from the trial at any time. Participants will also be asked to share relevant data with people from the investigation. Once the nurse has received the information, he or she will notify the technician to record and mark the blood products for further work.

On the consent form, participants will be asked if they agree to the use of their data. They can choose to withdraw from the trial. Participants will also be asked for permission for the research team to share relevant data with people from the investigators taking part in the research. This research does not involve collecting biological specimens for storage.

Ethics approval of this study will be granted by the Institutional Review Board of the medical centers: Shaanxi Province Corps Hospital of the Chinese People’s Armed Police Forces, Third Affiliated Hospital of Guangxi Medical University, and the 908th Hospital of Chinese People’s Liberation Army Joint Logistic Support Force. Any modification of protocol must be agreed on and authorized by a Research Committee.

Personal information about potential and enrolled participants will be collected, shared, and maintained only in this research group. Personal information will be protected confidentially before, during, and after the RCT. No conflicts of commercial interests are present, regardless of the conclusions.

### Choice of comparators

Participants receiving transfusion of blood components prepared from median-age male donors (25–45 years) will be grouped as the control in the investigation. The definition of control is nearly the same as previously designated in retrospective investigations in Canada [5] and Scandinavia [3]. It may minimize the potential differential effects of younger donors (< 25 years).

### Randomization and blinding design

This study will use a stratified block randomization method. The trial includes three groups divided by blood donor age: < 25, 25–45, and > 45 years. The length of each block is six. Screened patients will be assigned a screening number according to the order of admission. Patients who meet the inclusion criteria and have signed the informed consent form will be assigned a subject identification number, which consists of “S,” the block number and the order number in the block. For example, the identification number of the first patient in the first block will be “S001–1”. Blood components will be prepared as follows: sex and age information of the donors will be provided to the transfusion departments of the three study centers by the aforementioned blood centers, together with the blood components and with the expiration date and the ABO and Rh blood types. Other personal details will not yet be known to the investigators, such as name and ID card number. Blood components from male donors of different ages (< 25, 25–45, and > 45 years) will be reserved separately at the specified location and allocated following a stratified block randomization method. In short, the blood components will be labeled with the block number and the random number by a trained investigator of the Department of Blood Transfusion in the centers, and this investigator will not allocate the blood components so as to conceal the sequence until the interventions are assigned. The patients will then be transfused with blood components by matching the last number of the subject identification number with the random number of the blood components in the given block. For example, the first patient enrolled in the first block will be transfused with the blood components with the random number of “1” in the first block of blood components.

The allocation sequence will be generated by a biostatistician who will not be involved in the enrollment of participants directly. The investigators of the three study centers will enroll participants following the inclusion and exclusion criteria. A trained investigator of the Department of Blood Transfusion in the centers will allocate the blood components. Because our sample size estimates have taken into account the loss to follow-up, this study will not supplement patients who failed to randomize.

Participants, surgeons, and designers will be blinded to DAIP. Only designated investigators who are responsible for allocating blood to SICU patients are permitted to be unblinded. The outcome will be assessed blindly by a biostatistician who is blinded for the random plan. After data analysis, the results of the age grouping of the blood components will be unblinded by the biostatistician who prepares the random plan.

### Study procedure, data collection, and management

Each medical center will designate one or two investigators to gather patient baseline, outcome, and other trial data by telephone interview, face-to-face communication, and/or electronic medical record. Two electronic files—Transfusion Research and Case Report Form (CRF)—will be created in advance. Patient enrollment, research progress, and relevant hospitalization information will be stored. All data will be entered into the CRF first, after which double data entry will be performed. All electronic data will be stored by the designated investigator, and the paper-based materials of each patient will be collected together.

The coordinating center, steering committee, endpoint adjudication committee, and data management team has been established for coordination and cooperation concerning personnel or blood supply, supervision and guidance for trial execution, discontinuing and endpoint judgment, and data management, respectively. The coordinating center is composed of the study sponsor, Xianfei Zeng, and the directors of the Department of Blood Transfusion in the centers, Xingbin Hu, Yan Liao, and Dongjian Wang. The trial steering committee and endpoint adjudication committee are composed of Xianfei Zeng, Xingbin Hu, and Zhijun Tan, who will attend a specialized web conference bimonthly for supervising and overseeing the conduct and progress. The data management team is composed of Xingbin Hu, Xiaoshuang Wu, and Zhijun Tan; this team is independent from the sponsor and has no competing interests. All raw data from the three centers will be directly submitted to the data management team for statistical processing. Additionally, the principal investigators (PIs) of the Department of Blood Transfusion in the centers are responsible for all aspects of local organization including identifying potential recruits and taking consent.

Several strategies exist to improve adherence to intervention protocols. First, investigators at centers should describe the potential benefits from the DAIP investigation clearly so they are readily understood by patients or their first-degree relatives, such as mortality risk being reduced by transfusion with blood components from a male, which was designed in this study. Second, in consideration of the uncertainty of blood transfusion for emergency patients, at least two designated investigators are responsible for blood allocation at every center in order to ensure the carrying out around the clock of the investigation. Third, a supervisor will retrospectively review the cases for monitoring adherence once a month.

The interim analysis will be performed for quality control, the results of which cannot be used to determine premature stopping. No problems are anticipated that will be detrimental to the participants. Because the blood components from females who have experienced pregnancy increase the transfusion-related acute lung injury (TRALI) risk of blood recipients, the participants in the DAIP investigation may benefit from the transfusions of blood from male donors.

### The criteria for discontinuing

When the framed age donor’s blood is not available for clinically emergency use following the randomization process, the designed blood allocation principle will not be appropriate for the enrolled patient. The blood components will be transfused for life saving arbitrarily, regardless of the age of the donors. The cases will be discontinued.

The blood transfusion reactions, including nonhemolytic febrile transfusion reaction, transfusion-associated anaphylaxis, hemolytic transfusion reaction, and transfusion-associated circulatory overload, and so on, will be reported timely to the Transfusion Services Committee established in every research center. Such reporting cannot be completely eradicated following the current regulations or standards for blood transfusion therapy and therefore cannot affect the progress of the DAIP study.

### Patient follow-up

During the 28-day observation period, if the recruited patients survived and left the hospital, a follow-up will be conducted to record the survival time. The follow-up will be terminated after 28 days. For post-trial care, no harm is anticipated, and no compensation is being offered for trial participation.

### Protocol amendments

Any necessary changes made to the plan protocol from the sponsor will take place by notification from the sponsor to the centers, and a copy of the revised protocol will be sent to the principal investigator (PI) to be added to the Investigator Site File. In addition, we will update the protocol with the changes on the clinical trial registry website.

### Auditing the trial conduct

The ethical review will be carried out before and after DAIP investigation by institutional review board of the medical centers. The trial steering committee should hold a specialized meeting or web conference bimonthly for supervising the conduct and progress until reaching the sample size The independent data management team will review and improve the quality of data once a month.

### Dissemination policy of the current research

The results from the current study will be published and the data used only for research, without leaking any patients’ personal information. In addition, data are not permitted to be used for commercial purposes. Thus, the results will be communicated to any relevant groups only via publications.

### Statistics analysis

Statistical analyses will be performed with SPSS (version 19.0) and GraphPad Prism 5 (GraphPad Software) for Windows. A *p* value < 0.05 will be considered statistically significant. Continuous variables will be presented as the mean ± SD or median (25th and 75th percentiles), and categorical data will be expressed as percentages. A student’s *t*-test, the Mann–Whitney *U* test, or the Kruskal–Wallis test, as appropriate, will be used to compare the means or medians of continuous variables between or among groups. Categorical data will be tested using a chi-square test. Kaplan–Meier analysis, two-way ANOVA, and Cox proportional hazard models will be used to describe the survival characteristics, determine any interactions between variables, and explore potential factors promoting the death of SICU patients, respectively.

In this study, the missing data will not be imputed, and sensitivity analysis will be made to evaluate the effects of the missing data. Patients who are randomized to the intervention but do not adhere to it will not be included in the full analysis set.

## Discussion

Much research on aging has developed in recent years. Strategies to lessen the effects of aging include signal pathway intervention, key gene modification, exercise, and dietary control, and these have been administered in animals or humans [18]. In addition, a study found that proteins derived from younger individuals could fight against aging [8], suggesting that younger blood may benefit patients. In fact, blood transfusions have attracted attention as an antisenescence strategy because this classic therapy could potentially distribute blood components from younger donors, as necessary. Unfortunately, few data are available on the effects of blood transfusions from younger donors on the prognosis of severely ill patients [3, 5, 15, 16]. Consequently, most of the published data are from retrospective studies [19]. In the current DAIP protocol, we designed a novel and large-sample clinical trial, which will be conducted with randomization and a multicenter model. The benefits of this protocol include defined eligibility criteria, a definition of the 28-day survival primary outcomes, and a description of statistical analysis methods. Overall, the DAIP protocol will provide rigorous data on the prognosis of severely ill patients after blood transfusion from young donors.

Blood transfusion is a common therapy in hospitals, particularly for serious trauma and major surgeries [20, 21]. However, the prognosis can differ even if the same blood transfusion strategy is applied. More than one group has speculated that blood components that differ in storage time, donor sex, and donor age can lead to inconsistent therapy efficacy [17, 22, 23]. Although laboratory evidence suggests that younger blood components are superior to older blood components [7, 8, 13, 18], more direct evidence in humans is urgently needed. We conceived the DAIP protocol, which is an RCT with a blinded design. We believe this protocol will lead to positive feedback in terms of transfusion medicine and gerontology for the following reasons. First, the findings from the DAIP research will reduce confusion about the differential effects of administering blood components from young or old donors. Second, if blood components from younger individuals can indeed benefit recipient patients, practitioners will have more confidence in conducting blood transfusions accurately, which would include screening donors according to age. Finally, positive conclusions from the current protocol may change the allocation principle in blood administration.

In contrast to other controlled trials, conducting blood transfusion clinical research has unique challenges. Blood components are well known to have a limited shelf life in comparison with chemical drugs, which imposes great challenges for RCTs. We will employ a stratified block randomization method in the DAIP study to avoid this problem. This randomized method is also feasible, as we have collected data from 60 cases using this approach. Another problem is that patients in the SICU tend to need massive transfusion, and consequently, such need will increase the difficulty in using the framed age donor’s blood [24, 25]. If a patient requires a massive transfusion while the framed age donor’s blood is not available, we will have to exclude this case from the DAIP investigation. Finally, patients in the SICU are more likely to use several blood products including red blood cells, plasma, and platelets [26]. Since these patients may require different treatments that may have multiple effects on the study, we designed our statistical methods to account for multiple possibilities and possible redundancies. At least two critical factors should be considered when interpreting previous retrospective investigations. First, the donor sex is a confounding factor and should be taken into account in analyses [1, 27]. Second, the most frequently referenced study enrolled patients who were transfused with more than one unit of red blood cells [19]. This approach is problematic because the influence of donor age on patient outcome may have been obscured by the effects of blood dosage. Given these limitations, we tried to exclude donor sex bias to make a prospective, multicenter RCT study in DAIP.

Although we attempted to design a feasible and rational RCT protocol in DAIP, some questions remain. In contrast to reported similar investigations [28, 29], all of the current protocol’s recruited patients will be from the SICU, and these patients cannot represent patients who are not undergoing surgery. Thus, if possible, further design and research will be necessary to include other patients. Another issue concerns the disease spectrum. Different trauma types involve different organ and tissue damage, which may exhibit unexpected responses to blood transfusion [30]. In the DAIP protocol design, we did not take this into account because of the complicated statistics required. However, in our next study, we will emphasize a single disease analysis based on DAIP research. Storage-associated damages to blood products is an active topic in transfusion medicine, and storage time is not a key factor in prognosis [31–33]; hence, we did not consider this factor. However, possibly, differences resulting from storage damage will still exist when employing the DAIP protocol.

After the DAIP study is concluded, the collected data will indicate whether blood products from younger donors can benefit recipient patients. We believe that these DAIP data resulting from clinical research will be helpful in promoting exploration of this mechanism in laboratory medicine. The present protocol, a novel proposal to conduct RCTs on blood products from different age donors, is relevant to transfusion medicine, trauma management, and gerontology.

## Trial status

The protocol amendment number is DAIP 3.0 and was issued on 22 March 2019. The first recruitment began on 1 April 2019. The approximate date when recruitment will be completed is March 2021.

## Supplementary information

**Additional file 1.**

## Data Availability

The datasets obtained and/or analyzed during the current study are available from the corresponding author on reasonable request.

## Abbreviations

RCT: Randomized controlled trial
ChiCTR: Chinese Clinical Trial Registry
GDF-11: Plasma protein growth differentiation factor 11
MANF: Mesencephalic astrocyte-derived neurotrophic factor
DAIP: Donor age influence on prognosis
SICU: Surgical intensive care unit
APACHE-II: Acute Physiology and Chronic Health Evaluation II
SOFA: Sequential Organ Failure Assessment
CRF: Case report form
TRALI: Transfusion-related acute lung injury
